# Supplementary material for: PD-L1-Targeted Co-Delivery of Two Chemotherapeutics for Efficient Suppression of Skin Cancer Growth
Source: Pharmaceutics. 2022 Jul 18;14(7):1488. doi: 10.3390/pharmaceutics14071488 (PMC9318418; doi:10.3390/pharmaceutics14071488)
Supplement: Supplementary file 1 [file pharmaceutics-14-01488-s001.zip › pharmaceutics-1793164-supplementary.pdf]

Additional file 1

PD-L1-targeted co-delivery of two chemotherapeutics for synergistic suppression of skin cancer in vitro and in vivo

Fatemeh Movahedi, Jie Liu, Bing Sun, Pei Cao, Luyao Sun, Christopher Howard, Wenyi Gu, and Zhi Ping Xu*

Australian Institute for Bioengineering and Nanotechnology (AIBN), The University of Queensland, St Lucia, QLD 4072, Australia

* Corresponding authors. Email: gordonxu@uq.edu.au; Tel: 61 7 33463809

Determination of the number of conjugates

Briefly, the total number of lipid molecules in the outer lipid layer of each LCP was estimated by Equation 1:

$$N_{lip}=\frac{4\pi(r+h)^2}{a} \qquad \text{(Equation 1)}$$

where r is the radius of CaP core, h is the thickness of lipid layer (taken as 5 nm) and *a* is the average area per lipid molecule (0.7 nm^2 for DOPC). Then, the number of LCP NPs per ml was calculated by Equation 2:

$$N_{\mathrm{LCP}}=\frac{\mathrm{Clip}\times \mathrm{NA}}{Nlip\times 1000} \qquad \text{(Equation 2)}$$

where C_{lip} is the molar concentration of DOPC, and NA is the Avogadro number. Finally, the number of PD-L1 antibody of folic acid ligands per LCP NP were determined by Equation 3:

$$N_{\mathrm{lig}}=\frac{\mathrm{Clig}\times \mathrm{Nlip}}{Clip} \qquad \text{(Equation 3)}$$

where C_{lig} is the molar concentration of the ligand.

Table S1. List of synthesised NPs

Nanoparticle code	Payload	Number of FA per NP	Number of PD-L1 per NP
OTS-ABZ-LCP	ABZ and OTS	0	0
Cy5-LCP	Cy5 dsDNA	0	0
Cy5-LCP-P40	Cy5 dsDNA	0	40
Cy5-LCP-P80	Cy5 dsDNA	0	80
Cy5-LCP-P160	Cy5 dsDNA	0	160
OTS-ABZ-LCP-P40	ABZ and OTS	0	40
OTS-ABZ-LCP-P80	ABZ and OTS	0	80
OTS-ABZ-LCP-P160	ABZ and OTS	0	160
OTS-ABZ-LCP-F50	ABZ and OTS	50	0
OTS-ABZ-LCP-F100	ABZ and OTS	100	0
OTS-ABZ-LCP-F200	ABZ and OTS	200	0
OTS-ABZ-LCP-F50P40	ABZ and OTS	50	40
OTS-ABZ-LCP-F50P80	ABZ and OTS	50	80
OTS-ABZ-LCP-F50P160	ABZ and OTS	50	160
OTS-ABZ-LCP-F100P40	ABZ and OTS	100	40
OTS-ABZ-LCP-F100P80	ABZ and OTS	100	80
OTS-ABZ-LCP-F100P160	ABZ and OTS	100	160

Table S2. The mean size of PD-L1/folic acid conjugated OTS-ABZ-LCPs

Sample name	Number mean size (nm)	Sample name	Number mean size (nm)
OTS-ABZ-LCP-F50	57.3	OTS-ABZ-LCP-F100P40	66.0
OTS-ABZ-LCP-F100	59.7	OTS-ABZ-LCP-F100P80	65.5
OTS-ABZ-LCP-F200	59.3	OTS-ABZ-LCP-F100P160	65.3
OTS-ABZ-LCP-P40	58.7	OTS-ABZ-LCP-F50P40	64.7
OTS-ABZ-LCP-P80	61.7	OTS-ABZ-LCP-F50P80	62.4
OTS-ABZ-LCP-P160	63.1	OTS-ABZ-LCP-F50P160	63.2
OTS-ABZ-LCP	58.0		

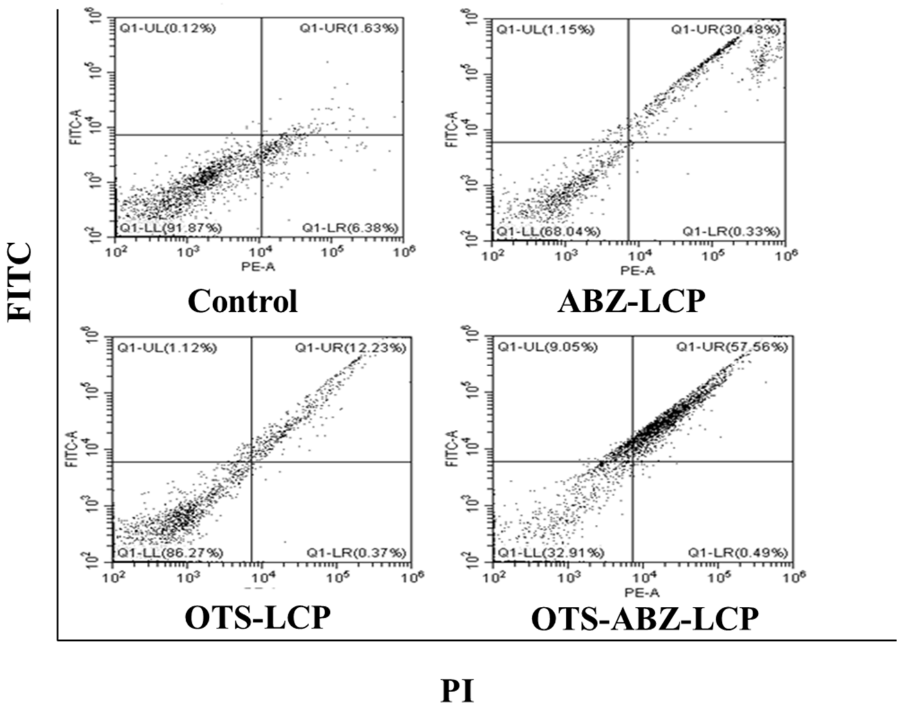

Figure S1. Annexin V-FITC/PI double staining analysis of apoptosis in B16F0 cells treated with ABZ-LCP, OTS-LCP and OTS-ABZ-LCP for 24 h (Annexin V and PI negative cells were considered as live, Annexin V positive and PI negative as early apoptotic, Annexin V and PI positive as late apoptotic and Annexin V negative and PI positive as necrotic cells).

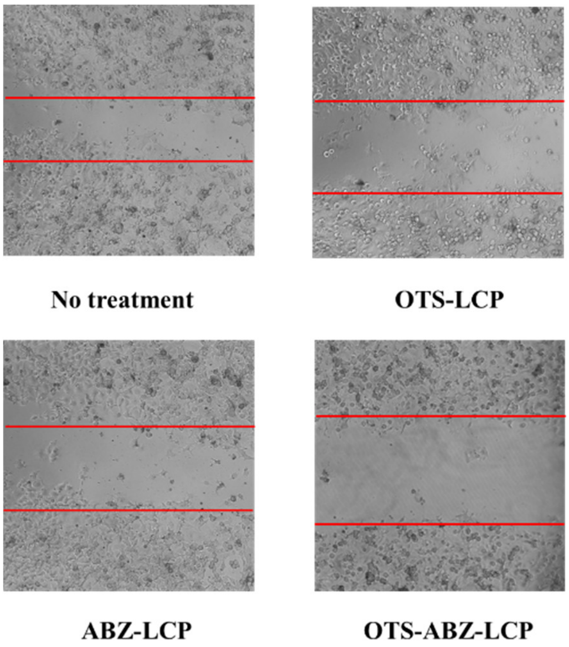

Figure S2. Relative migrated number of cells determined by wound healing assay for B16F0 cells treated with LCP formulations for 4 h. The equivalent concentration of ABZ and OTS was 2.5 μg/ml and 64 ng/ml, respectively.

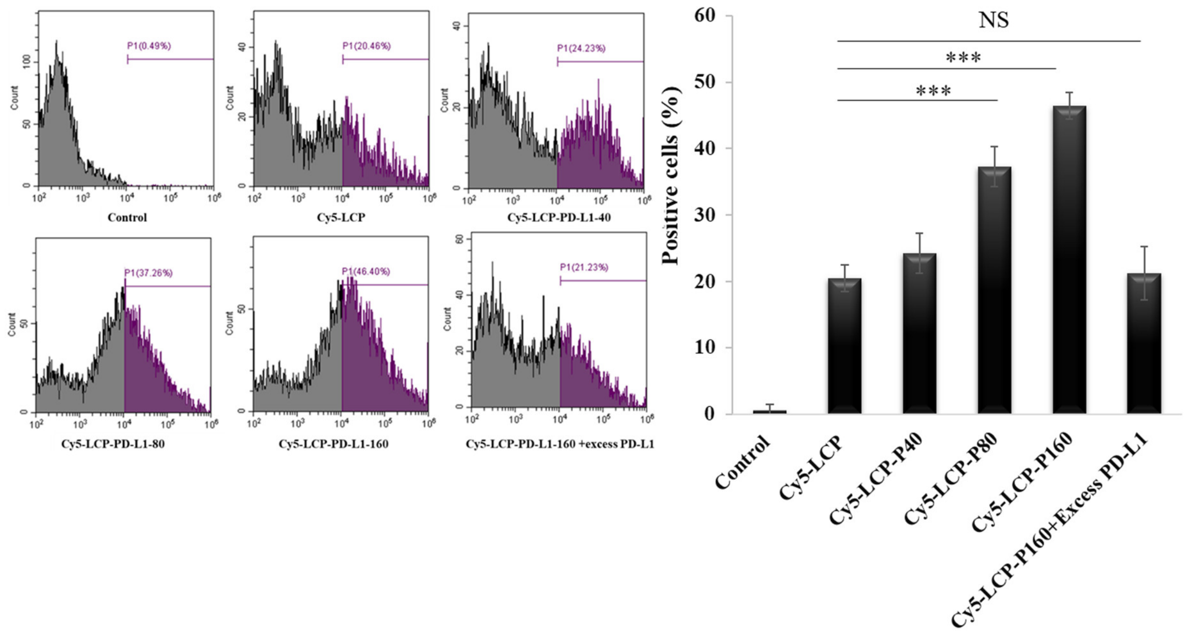

Figure S3. Cellular uptake of Cy5 dsDNA-loaded LCPs conjugated with 40, 80 or 160 PD-L1 antibodies per NP by B16F0 cells after 4 h.

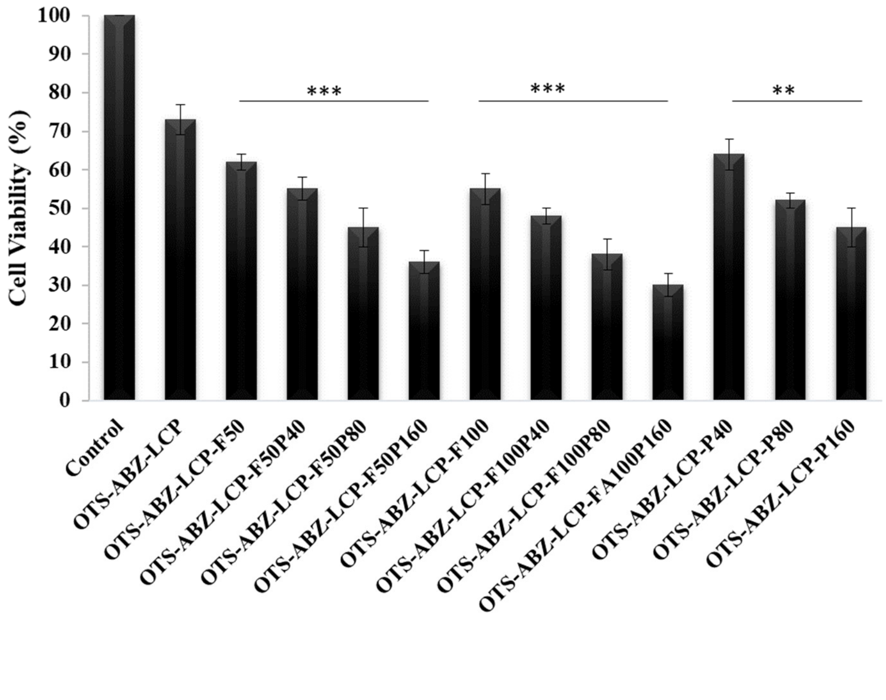

Figure S4. Cytotoxic effect of OTS-ABZ-LCP NPs dual conjugated with different numbers of folic acid and PD-L1 antibody (ABZ: 100 ng/ml; OTS964: 50 ng/ml).

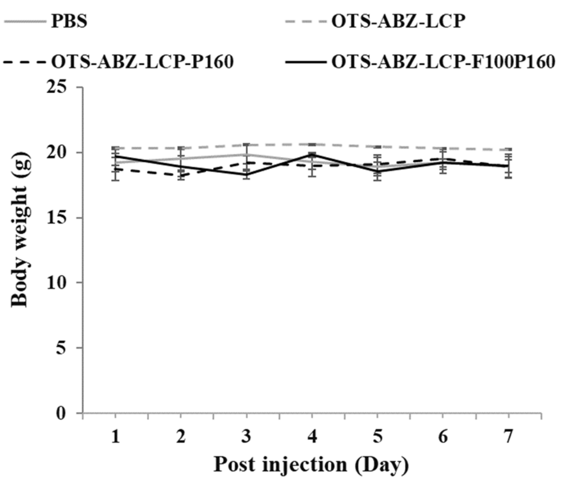

Figure S5. The average body weight of B16F0-bearing mouse treated with OTS-ABZ-LCP, OTS-ABZ-LCP-P160 and OTS-ABZ-LCP-F100P160 (equivalent amount of albendazole 2.5 mg/kg and 1 mg/kg OTS in all formulations) intraperitoneally injected 3 times every two days compared to that of PBS injection

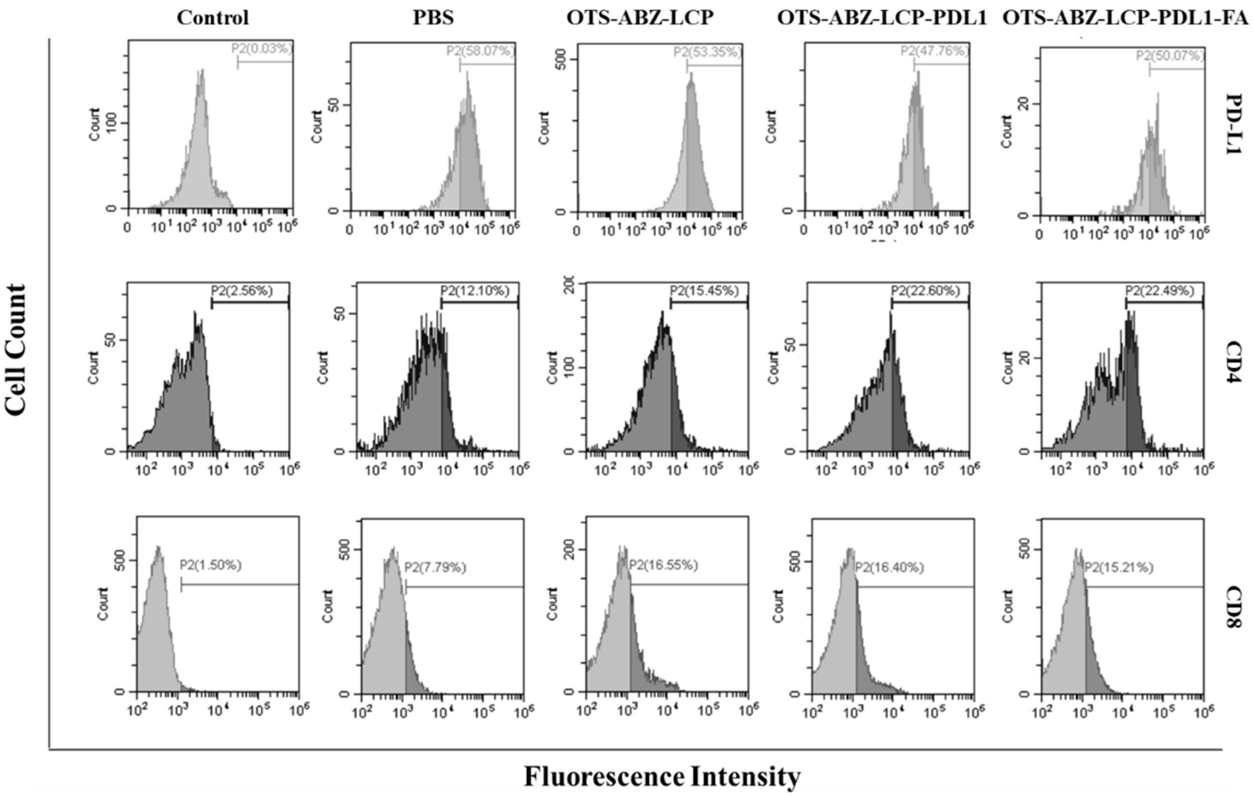

Figure S6. Flow cytometric analysis of PD-L1 expression, CD4^{+} and CD8^{+} in tumour population.

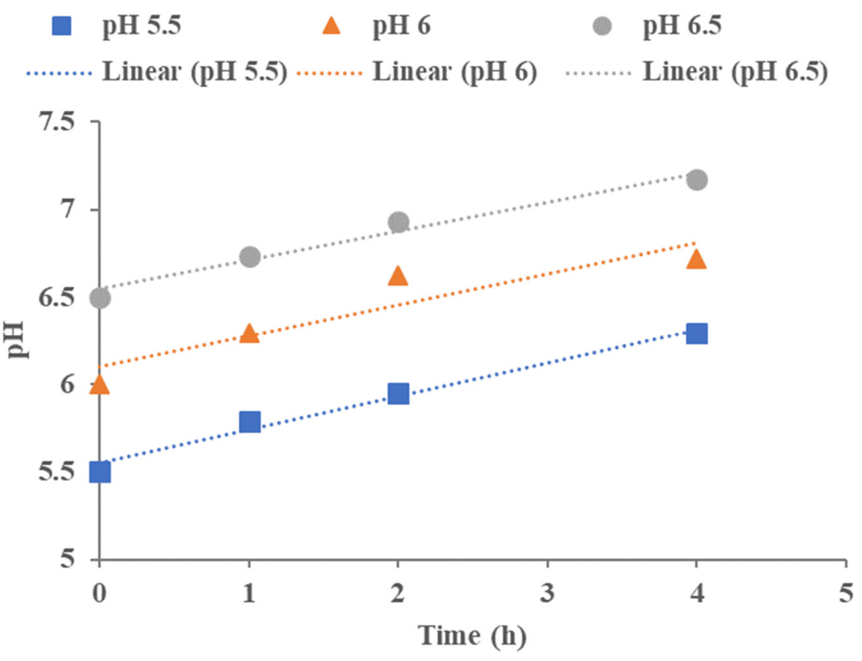

Figure S7. pH alteration by degradation of LCP NPs in DI water with set pH at 5.5, 6 and 6.5
